# Laminar flow reduces cases of surgical site infections in vascular patients

**DOI:** 10.1308/003588413X13511609956011

**Published:** 2013-01

**Authors:** DC Bosanquet, CN Jones, N Gill, P Jarvis, MH Lewis

**Affiliations:** ^1^Cwm Taf Health Board,UK; ^2^University of Glamorgan,UK

**Keywords:** Vascular surgical procedures, Surgical wound infection, Laminar air-flow areas, Postoperative complications

## Abstract

**Introduction:**

Numerous strategies are employed routinely in an effort to lower rates of surgical site infections (SSIs). A laminar flow theatre environment is generally used during orthopaedic surgery to reduce rates of SSIs. Its role in vascular surgery, especially when arterial bypass grafts are used, is unknown.

**Methods:**

A retrospective review of a prospectively maintained database was undertaken for all vascular procedures performed by a single consultant over a one-year period. Cases were performed, via random allocation, in either a laminar or non-laminar flow theatre environment. Demographic data, operative data and evidence of postoperative SSIs were noted. A separate subgroup analysis was undertaken for patients requiring an arterial bypass graft. Univariate and multivariate logistical regression was undertaken to identify significant factors associated with SSIs.

**Results:**

Overall, 170 procedures were analysed. Presence of a groin incision, insertion of an arterial graft and a non-laminar flow theatre were shown to be predictive of SSIs in this cohort. In the subgroup receiving arterial grafts, only a non-laminar flow theatre environment was shown to be predictive of an SSI.

**Conclusions:**

This study suggests that laminar flow may reduce incidences of SSI, especially in the subgroup of patients receiving arterial grafts.

Infection is a major potential complication of bypass grafting in vascular surgery. The incidence of vascular graft infections ranges from 1% to 10% of cases.^1–4^ However, a more commonly accepted infection rate is considered to be 2–3%.^4^ The sequelae of graft infections can be catastrophic. Aortic graft infections carry a mortality of 45–70%^2^ and graft infection in general leads to prolonged in-hospital stay, reoperation and, occasionally, limb amputation. Even nongraft related surgical wound infections result in significant morbidity. Length of in-hospital stay was increased by an average of 12.2 days following wound infections at an average cost of £3,313 for superficial wound infections and £5,347 for deep infections.^5^

A number of factors are associated with an increased risk of graft infection. These include groin incision,^1,2,4^ postoperative wound complication,^2^ diabetes mellitus,^1,6^ emergency surgery,^4^ prolonged hospital stay,^1^ smoking,^1^ advanced age (over 65 years)^1^ and early reoperation.^7^ The clinical presentation of graft infections is varied, with infrainguinal bypass graft infections presenting postoperatively after a mean of four months and aortic graft infections presenting after a mean of three years.^1^

Numerous strategies have been developed to reduce these infections, including antibiotic prophylaxis, meticulous aseptic technique and, more recently, antibiotic/antimicrobial containing graft materials.^1–9^ Laminar flow, a system that creates a homogenous flow of air in the operating room with very little turbulence, is used widely in orthopaedic procedures, especially during the insertion of prosthetic graft materials, to minimise contamination of the surgical field with airborne microbes.^10^ Surprisingly, the uptake of laminar flow in other surgical fields is limited. In spite of the similarity of operative principles during the insertion of prosthetic material in both orthopaedic and vascular surgery, and the potentially catastrophic risks of infection, there have been no studies looking at laminar flow specifically in vascular surgery.

We noted an apparent difference in a single team’s post-operative infection rates in patients undergoing surgery in non-laminar theatre environments compared with those who had undergone surgery in laminar flow theatres despite similar operating team and surgical practice. This appeared to be particularly in cases requiring arterial bypass surgery. We therefore undertook a review of the incidence of postoperative infections in patients who had vascular surgery in either laminar or non-laminar flow theatres and performed multiple logistic regression to identify factors influencing the rate of infection.

## Methods

A one-year retrospective analysis was carried out on a prospectively collected database of consecutive patients undergoing open vascular procedures (venous and arterial) performed by a single vascular surgeon. Endovascular or other minimally invasive vascular procedures were not included. Patients undergoing vascular procedures requiring arterial bypass using grafts were further analysed. Procedures were performed in both laminar flow and conventional theatre environments with allocation randomly assigned via the waiting list to one of three weekly scheduled lists (two half-day lists in a non-laminar flow theatre, one half-day list in a laminar flow theatre).

All procedures were performed by the same consultant surgeon, assisted by the same theatre staff and performed using similar surgical techniques. Skin preparation was identical, consisting of skin clipping immediately prior to skin preparation, which was with a chlorhexidine-alcohol solution. In the case of arterial bypass, conduit materials included autologous vein (used preferentially for infrainguinal bypasses), woven dacron or polytetrafluoroethylene. Prosthetic graft materials did not contain antimicrobial or antibiotic agents. Patients undergoing vascular procedures routinely received preoperative antibiotic prophylaxis at the time of induction (typically co-amoxiclav or a cephalosporin and metronidazole, depending on allergies).

Data collected included age, sex, procedure performed, operative time, use of a groin incision, predisposing factors for infection including diabetes mellitus and other comorbidities (defined as per international standards),^11–14^ current smoking status, graft type and evidence of postoperative surgical site infection (SSI). SSIs were identified, as supervised by one consultant microbiologist, by a review of the medical records for clear evidence of wound infection (Table 1) and graded as superficial (involving skin or subcutaneous tissue) or deep (involving muscle, fascia or graft material) according to Centers for Disease Control and Prevention criteria.^15^

**Table 1:** Criteria for surgical site infection

Clinical documentation of wound infection, ie: cellulitis erythema wound breakdown purulent discharge +/- microbiological evidence +/- pyrexia +/- increased inflammatory markers or white cell count

Initial statistical analysis to compare patient groups between the two theatres used a two-tailed Student’s t-test for continuous variables (after confirmation of normality with a Kolmogorov–Smirnov test) and a two-tailed chi-square or Fisher’s exact test for categorical variables as appropriate. Univariate analysis was undertaken to examine potential variables affecting infection rates. A stepwise (backward:LR method) multivariate logistical regression analysis was undertaken subsequently to identify significant factors affecting rates of SSIs. A similar regression analysis was carried out for the subgroup of patients requiring arterial bypass. The Hosmer–Lemeshow test was used to assess model goodness of fit throughout.^16^ *P*-values of <0.05 were considered statistically significant. SPSS^®^ version 16.0 (SPSS, Chicago, IL, US) was used for statistical analysis.

## Results

A total of 170 vascular operations were performed during the study period (114 in a non-laminar flow theatre, 56 in laminar flow), of which SSIs occurred in 23 patients (13.5%; 14 superficial infections, 9 deep infections). Details of patient data, operative data, significant co-morbidities and evidence of SSIs are given in Table 2. Patients in laminar flow theatres were more likely to have an arterial bypass procedure requiring a graft insertion and were generally (but not statistically significantly) older. Despite these data, SSIs tended to occur at a lower rate in the laminar flow theatre environment (7% vs 17%, *p*=0.1).

Infection rates appeared to be higher in patients undergoing arterial bypass using grafts and this subgroup was therefore analysed separately. Overall, 81 patients required an arterial graft as part of their operative procedure, 69 of which involved insertion of prosthetic graft material. The operations performed are detailed in Table 3. Patients in this subgroup were matched equally between theatres regarding baseline characteristics, operative details and comorbidities. Despite this, a significantly greater rate of SSIs were seen in the non-laminar flow theatre environment (11% vs 33%, *p*=0.034) and a significantly greater number of arterial graft infections developed requiring graft removal in the non-laminar flow theatre environment (0% vs 8%, *p*=0.0086) (Table 4).

These initial results had demonstrated a greater proportion of SSIs in patients undergoing surgery in a non-laminar flow theatre environment, which was significant in the subgroup requiring insertion of arterial grafts. Univariate and multivariate analysis was used to examine which factors were significantly associated with a greater risk of SSIs among all vascular cases (Table 5). On univariate analysis, male sex, a longer operative time, presence of a groin incision and insertion of arterial grafts were shown to predict SSIs. Following multivariate analysis (Hosmer–Lemeshow test, *p*=0.641), male sex and operative time lost their significance but the use of arterial grafts, the presence of a groin incision and the use of a non-laminar flow theatre were shown to increase the odds of developing an SSI.

Univariate and multivariate analysis was subsequently performed on the subgroup receiving arterial grafts. As shown in Table 6, the use of a non-laminar flow theatre was the only independent predictor for wound infections, significant at both univariate and multivariate analysis (Hosmer–Lemeshow test, *p*=0.747).

**Table 2:** Patient data from laminar and non-laminar flow theatres

	Non-laminar flow	Laminar flow	*P*-value
Total	114	56	
Sex (M:F)	76:38	33:23	0.323*
Mean age (range)	64.8 (19–87)	69.1 (19–90)	0.06^‡^
Mean operative time (range) in minutes	85.1 (20–350)	95.5 (20–225)	0.263^‡^
‘Re-do’ procedures	8 (7%)	4 (7%)	1.0^†^
Arterial procedures	92 (81%)	51 (91%)	0.102*
Arterial grafts used	46 (40%)	35 (63%)	0.007*
Groin incision	50 (44%)	22 (39%)	0.539*
Antibiotic prophylaxis given	93 (82%)	50 (89%)	0.283*
Diabetes mellitus	15 (13%)	4 (7%)	0.306^†^
Ischaemic heart disease	36 (32%)	13 (23%)	0.258*
Chronic obstructive pulmonary disease	18 (16%)	9 (16%)	0.962*
Surgical site infections	19 (17%)	4 (7%)	0.1^†^

* chi-square test;

† Fisher’s exact test;

‡ Student’s t-test

**Table 3:** Operations performed involving arterial bypass using grafts

Surgical procedure	Laminar flow theatre	Non-laminar flow theatre	Total
AAA repair (open) / aortic procedures	23	22	45
Infrainguinal bypass	11 (of which 6 vein grafts were used)	17 (of which 6 vein grafts were used)	28
Axillofemoral / axilloaxillary bypass	0	5	5
Other procedures	1	2	3
**Total**	**35**	**46**	**81**

AAA = abdominal aortic aneurysm

**Table 4:** Details of patients undergoing graft insertion

	Non-laminar flow	Laminar flow	*P*-value
Total patents	46	35	
Sex (M:F)	36:10	25:10	0.48*
Mean age (range)	67.4 (19–87)	70.7 (37–83)	0.213^‡^
Mean operative time (range) in minutes	126.4 (30–350)	120.0 (25–225)	0.588^‡^
‘Re-do’ procedures	4 (9%)	2 (6%)	0.694^†^
Venous conduit used	6 (13%)	6 (17%)	0.8424*
Groin incision	22 (48%)	16 (46%)	0.778*
Antibiotic prophylaxis given	44 (96%)	35 (100%)	1.0*
Arterial graft infection (requiring graft removal)	9 (8%)	0 (0%)	**0.0086** ^†^
Diabetes mellitus	7 (15%)	4 (11%)	0.749^†^
Ischaemic heart disease	21 (46%)	10 (29%)	0.117*
Chronic obstructive pulmonary disease	11 (24%)	8 (23%)	0.912*
Surgical site infections	15 (33%)	4 (11%)	**0.034** ^†^

* chi-square test;

† Fisher’s exact test;

‡ Student’s t-test

**Table 5:** Univariate and multivariate analysis for factors associated with an increased risk of developing a surgical site infection among all vascular cases

	SSI	No SSI	Univariate analysis *p*-value	Multivariate analysis *p*-value	Odds ratio (95% CI)
Total	23	147			
Sex (M:F)	21:2	88:59	**0.008**	–	
Mean age (range)	67.7 (50–83)	66.0 (19–90)	0.787	–	
Mean operative time (range) in minutes	116.9 (35–230)	84.4 (20–350)	**0.011**	–	
‘Re-do’ procedures	3 (13%)	9 (6%)	0.512	–	
Arterial procedures	20 (87%)	123 (84%)	0.49	–	
Arterial grafts used	19 (83%)	62 (42%)	**0.001**	**0.002**	6.945 (2.092–23.056)
Groin incision	16 (70%)	56 (38%)	**0.004**	**0.013**	3.809 (1.319–10.994)
Antibiotic prophylaxis given	21 (91%)	122 (83%)	0.139	–	
Diabetes	4 (17%)	15 (10%)	0.282	–	
Ischaemic heart disease	10 (43%)	39 (27%)	0.320	–	
Chronic obstructive pulmonary disease	4 (17%)	23 (16%)	0.72	–	
Cases in non-laminar flow theatre	19 (83%)	95 (65%)	0.108	**0.026**	4.016 (1.178–13.689)

SSI = surgical site infection; CI = confidence interval

**Table 6:** Univariate and multivariate analysis for factors associated with an increased risk of developing a surgical site infection in cases receiving arterial grafts

	SSI	No SSI	Univariate analysis *p*-value	Multivariate analysis *p*-value	Odds ratio (95% CI)
Total	19	62		
Sex (M:F)	17:2	44:18	0.162	–	
Mean age (range)	68.6 (50–83)	68.9 (19–87)	0.676	–
Mean operative time (range) in minutes	124.7 (35–230)	123.2 (23–350)	0.913	–	
‘Re-do’ procedures	3 (16%)	3 (5%)	0.318	–
Groin incision	12 (63%)	26 (42%)	0.119	–	
Antibiotic prophylaxis given	18 (95%)	61 (98%)	0.592	–
Diabetes	4 (21%)	7 (11%)	0.217	–	
Ischaemic heart disease	9 (47%)	22 (35%)	0.735	–	
Chronic obstructive pulmonary disease	3 (16%)	16 (26%)	0.527	–	
Cases in non-laminar flow theatre	15 (79%)	31 (50%)	**0.04**	**0.047**	3.474 (1.016–11.883)

SSI = surgical site infection; CI = confidence interval

## Discussion

Laminar flow ventilation was first pioneered by Charnley in the 1960s to prevent ‘bacteriological contamination in the air of the operating theatre’.^17^ In his series of 455 patients undergoing hip arthroplasties, there was an infection rate of 9.5% in non-laminar flow theatres compared with 1.1% in laminar flow theatres.^18^ More recent studies have continued to show the importance of laminar flow in lowering postoperative SSIs.^10^ However, other studies suggest that laminar flow makes little difference to rates of SSIs^19,20^ or that it may even increase rates of infection.^21^ Despite this, theatres are generally all laminar flow as standard in newly built hospitals. Surprisingly, there have been no previous studies investigating the effect of laminar flow on SSI rates in vascular surgery.

In our series of 170 patients, 23 developed an SSI, 19 of which occurred in cases where arterial grafts were required. In this cohort, a greater proportion of cases came from the non-laminar flow theatre environment (33% vs 11%, *p*=0.034). Multiple logistic regression identified three factors associated with an increased risk of SSIs: the use of arterial grafts, a groin incision and the use of a non-laminar flow theatre. Insertion of arterial grafts and groin incisions have been shown previously to increase the risk of SSIs in vascular surgery.^22,23^ Subsequent multiple logistic regression in patients receiving arterial grafts demonstrated that operating in a non-laminar flow environment remained the only significant risk factor for SSIs in this cohort.

Due to the retrospective nature of our study, it was not possible to include other variables associated with increased SSI rates, such as renal disease, increased body mass index, emergency surgery and a high ASA (American Society of Anesthesiologists) grade.^8^ Regardless, these data suggest laminar flow may be important in preventing SSIs in patients undergoing vascular surgery, especially in those receiving arterial grafts.

A perhaps surprisingly small number of SSIs (*n*=4) were recorded in patients undergoing vascular procedures without insertion of arterial grafts. Evidence of SSIs was collected in hospital and during subsequent hospital visits. Almost certainly, there was a greater true rate of infection, which was treated in the community setting. However, the assumption that these cases represent the more severe spectrum of SSIs implies these data are valid for reducing significant infections. We suggest this makes the results more important rather than less so.

A note of caution is required regarding the analysis of these data. The case mix is heterogeneous and not equally matched between the two theatre environments. For example, all five axillary bypass procedures were undertaken in a non-laminar flow theatre although a greater percentage of arterial bypass procedures was performed in a laminar flow theatre. Administration rates of antibiotic prophylaxis were not identical between the two groups. Furthermore, retrospective SSI data collection lacks the sensitivity of prospective data collection. Given this, definitive data from a prospective randomised study of homogenous patients undergoing similar procedures with equivalent antibiotic prophylaxis in these two theatre environments are warranted before definitive conclusions can be drawn.

## Conclusions

The data from our study suggest that laminar flow may play an important role in reducing the incidences of SSIs in vascular surgery. This appears to be particularly so with operations involving arterial bypass using grafts.
